# Characterization and Flame-Retardant Properties of Cobalt-Coordinated Cyclic Phosphonitrile in Thermoplastic Polyurethane Composites

**DOI:** 10.3390/molecules29081869

**Published:** 2024-04-19

**Authors:** Xiangcong Zeng, Zhi Xu, Haoxun Li, Yun Xiong, Yigang Ding, Lili Xu, Shengpeng Liu

**Affiliations:** 1 School of Chemical Engineering and Pharmacy, Wuhan Institute of Technology, Wuhan 430205, China; 2 School of Technology Materials Science and Engineering, Wuhan Institute of Technology, Wuhan 430205, China

**Keywords:** hydrothermal, cobalt coordinated, limiting oxygen index, organophosphorus, metal-based cyclic phosphonitrile

## Abstract

Halogen-free organophosphorus flame retardants have promising application prospects due to their excellent safety and environmental protection properties. A cobalt-coordinated cyclic phosphonitrile flame retardant (Co@CPA) was synthesized via a hydrothermal method using hexachlorocyclotriphosphonitrile (HCCP), 5-amino-tetrazolium (5-AT), and cobalt nitrate hexahydrate (Co(NO_3_)2∙6H_2_O) as starting materials. The structure was characterized using Fourier transform infrared (FTIR), nuclear magnetic resonance spectroscopy (1H-NMR), scanning electron microscopy (SEM), and thermogravimetric analysis (TGA). Thermoplastic polyurethane (TPU) composites were prepared by incorporating 10-(2,5-dihydroxyphenyl)-9,10-dihydro-9-oxa-10-phosphame-10-oxide (ODOPB), Co@CPA, and silicon dioxide (SiO_2_) via melt blending. The flame-retardant performance and thermal stability of the TPU composites were evaluated through limiting oxygen index (LOI), vertical combustion (UL-94), TG, and cone calorimetric (CCT) tests. SEM and Raman spectroscopy were used to analyze the surface morphology and structure of the residual carbon. A synergistic flame-retardant effect of ODOPB and Co@CPA was observed, with the most effective flame retardancy achieved at a TPU:ODOPB:Co@CPA:SiO_2_ ratio of 75:16:8:1. This composition exhibited an LOI value of 26.5% and achieved a V-0 rating in the UL-94 test. Furthermore, compared to pure TPU, the composite showed reductions in total heat release, CO production, and CO_2_ production by 6.6%, 39.4%, and 48.9%, respectively. Our research findings suggest that Co@CPA demonstrates outstanding performance, with potential for further expansion in application areas. Different metal-based cyclic phosphonitrile compounds are significant in enriching phosphorus-based fine chemicals.

## 1. Introduction

Thermoplastic polyurethane (TPU), also referred to as polyurethane rubber, is classified under the thermoplastic elastomer (TPE) category [1,2,3]. Its notable characteristics include elasticity, wear resistance, chemical stability, and plasticity, which have led to its extensive utilization in industries such as aerospace, automotive, and marine [4,5,6]. However, TPU is an extremely flammable material, The molten droplets generated during the combustion process not only spread the fire but also release a significant amount of toxic and harmful gases, such as CO, HCN, NOx, etc. This poses a serious limitation on the application of TPU [7,8,9]. Inorganic flame retardants are commonly utilized in polymer for their non-flammable nature and high specific heat capacity, but their compatibility with TPU materials is lacking, necessitating the addition of large quantities, ultimately leading to a reduction in the mechanical properties of the polymer [10,11]. Halogen flame retardants offer excellent flame-retardant properties for TPU materials with minimal addition amounts, high compatibility, and no reduction in material mechanical properties. However, the combustion of halogenated flame retardants can produce various carcinogenic toxic substances like polybrominated dibenzo-p-dioxins and dibenzofurans, posing significant health risks [12,13].

In comparison, organophosphorus flame retardants exhibit high flame-retardant efficiency for TPU materials and good compatibility and produce non-toxic, pollution-free combustion by-products. These organophosphorus flame retardants have been extensively researched and widely utilized in flame-retardant TPU materials [14,15]. Cyclotriphosphazene and its derivatives, which are based on a phosphorus–nitrogen skeleton, exhibit remarkable thermal stability and structural diversity. The presence of the phosphorus–nitrogen skeleton in their structure allows for a conjugation effect, resulting in exceptional chemical stability and resistance to oxidation even after prolonged exposure to air [16,17,18,19,20]. Studies indicate that coordination compounds formed by combining these derivatives with metals not only display high thermal stability but also effectively suppress the release of heat and smoke during material combustion [21,22,23,24]. Metal elements can catalyze dehydration and chain breakage of the polymer matrix, leading to the formation of a stable carbon layer that effectively suppresses heat and smoke release during combustion. However, the poor compatibility between metal compounds and organic polymer materials results in inadequate dispersion of the former within the polymer matrix, leading to a deterioration in the mechanical properties of the polymer material and hindering the achievement of the desired flame-retardant performance [25,26,27,28,29]. As a result, researchers have recently turned their attention to metal–organic coordination compounds; these compounds, with organic ligands, not only exhibit excellent compatibility with polymers but also provide flame-retardant properties due to the flame-retardant elements present on the organic ligands [30,31,32,33]. Xilei Chen and colleagues utilized a copper metal–organic framework (MOF-Cu) for flame-retardant modification of thermoplastic polyurethane elastomer (TPU) composite materials. Their study revealed that the combined application of MOF-Cu with TPU resulted in a synergistic effect, leading to a substantial enhancement in flame-retardant performance. This study suggests that a composite material consisting of thermoplastic polyurethane (TPU), a copper metal–organic framework (MOF Cu), and ammonium polyphosphate (APP) exhibits the most effective flame-retardant properties with a limiting oxygen index (LOI) of 27%. Among these components, APP is a commonly used phosphorus-based flame retardant [34]. Therefore, the combination of metals with phosphorus-based flame retardants shows promising flame-retardant capabilities.

In this study, a metal coordination compound was synthesized by combining cobalt and cyclic phosphazene using a hydrothermal method. The compound’s structure and properties were analyzed through FTIR, 1H-NMR, and TG techniques. TPU composite materials were fabricated, and their flame-retardant properties, combustion behavior, and mechanical properties were investigated. The surface morphology and structure of the combustion products were also analyzed. The results indicate that the combination of cobalt with organic ligands in Co@CPA effectively suppressed the heat release and smoke release of composite materials. The successful modification of CPA with cobalt effectively reduced the fire risk and increased the safety of composite materials. These research findings will provide valuable guidance and reference for the widespread adoption of flame-retardant modification.

## 2. Experimental Section

### 2.1. Synthesis of Flame Retardants

**Figure 1:**
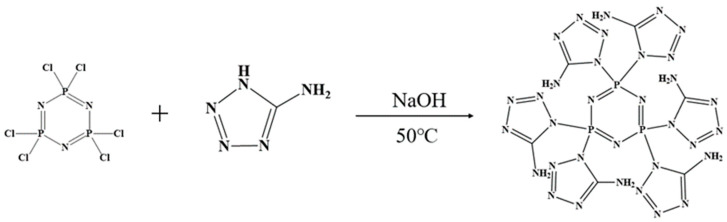
Synthetic route of CPA.

We weighed 0.8675 g (0.0025 mol) of hexachlorocyclotriphosphonitrile (HCCP) and dissolved it in 50 mL of acetonitrile. Next, we weighed 1.275 g (0.015 mol) of 5-amino-tetrazolium (5-AT) and dissolved it in 50 mL of deionized water. Both solutions were combined in a three-necked flask and stirred continuously at 50 °C until the solution became clear. Then, we added 0.6 g (0.015 mol) of solid NaOH and let the reaction proceed for 6 h. The resulting solution was rotary evaporated and vacuum-dried at 100 °C for 12 h. Subsequently, the methanol solution was stirred, filtered to separate NaCl solid, and then distilled to obtain the intermediate triphosphazene-co-5-aminotetrazolium (CPA). The CPA was vacuum-dried at 80 °C for 12 h. The synthesis route is illustrated in Figure 1.

**Figure 2:**
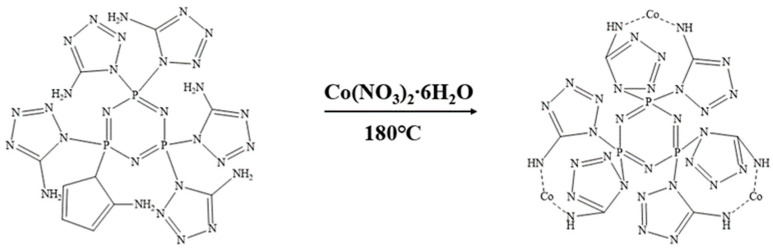
Synthetic route of Co@CPA.

We weighed 3.1956 g (0.005 mol) of CPA and 4.365 g (0.015 mol) of cobalt nitrate hexahydrate and placed them in a high-pressure reactor sealed with polytetrafluoroethylene. Next, we added 50 mL of deionized water to dissolve the mixture. Then, we weighed 1.2 g (0.03 mol) of NaOH solid and stirred it until the solution changed to an orange-red color. We introduced a specific amount of nitrogen gas and transferred the reactor to an electric constant-temperature drying oven. We allowed the reaction to proceed at 180 °C for 12 h, followed by centrifugation and three washes with deionized water. Finally, we vacuum-dried at 100 °C for 12 h to obtain the final product Co@CPA. The synthesis route is illustrated in Figure 2.

### 2.2. Preparation of TPU Composite Materials

TPU composite materials were prepared through physical melt blending. Initially, we dried TPU, ODOPB Co@CPA, and SiO_2_ for 12 h and set them aside. Due to the hygroscopic nature of Co@CPA, it should be promptly utilized. The detailed experimental procedure involved setting the temperature of the torque rheometer to 150 °C, adjusting the speed to 60 r/min, setting the temperature of the flat vulcanization machine to 160 °C, heating and mixing an appropriate amount of TPU for 3–4 min, adding ODOPB according to the formula in Table 1, and mixing Co@CPA, SiO_2_ for 10–15 min to ensure full melting of the flame retardant with TPU, resulting in TPU composite material. The composite material was then crushed with a grinder, evenly laid in a mold, and hot-pressed and shaped using a flat vulcanization machine for 10~15 min. After cooling to room temperature, the sample was removed, cut, and polished. Standard samples for vertical combustion, ultimate oxygen index, and cone calorimetry were prepared using the aforementioned methods. Mechanical performance test samples were prepared using an injection molding machine.

## 3. Results and Discussion

### 3.1. Characterization of CPA

**Figure 3:**
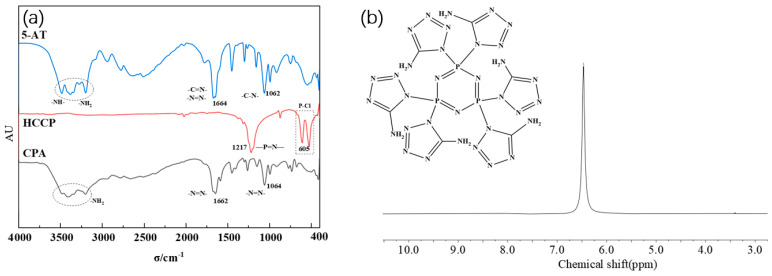
(**a**) FTIR spectra of CPA; (**b**) 1H NMR spectra of CPA.

As depicted in Figure 3a, 5-amino-tetrazolium (5-AT) exhibit characteristic peaks at -NH- and -NH_2_ absorption peaks between 3000 and 3500 cm^−1^, N=N and C=N absorption peaks at 1664 cm^−1^, and C-N absorption peaks at 1062 cm^−1^. The characteristic peaks of hexachlorocyclotriphosphonitrile (HCCP) remain almost unchanged. This allows for the determination of the structure of the intermediate CPA.

In Figure 3b, a unique set of absorption peaks at 6.4, corresponding to the absorption peak of H on -NH_2_, is observed at the chemical shift δ. This further confirms the substitution reaction between 5-AT and HCCP involving -NH- and P-Cl and supports the high purity of the synthesized intermediate CPA.

### 3.2. Characterization of Co@CPA

**Figure 4:**
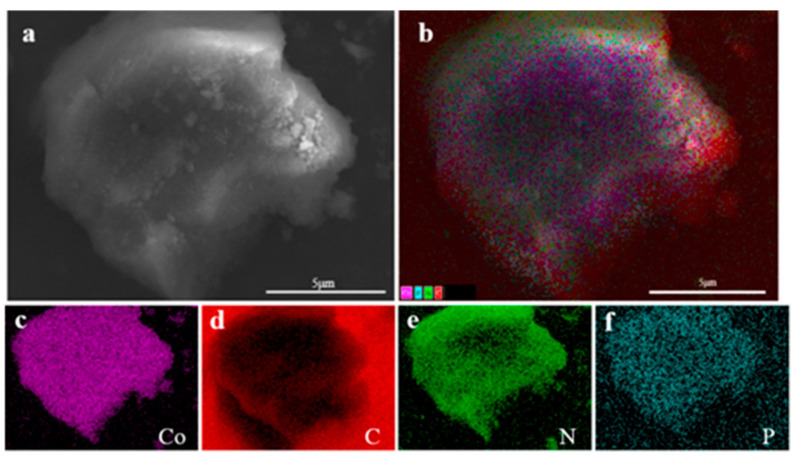
(**a**) The SEM overall view of Co@CPA (**b**) and EDS element layer image, (**c**) Co kα, (**d**) C kα, (**e**) N kα, (**f**) P kα.

Utilizing carbon flakes as a background, the sample Co@CPA was analyzed to examine specific areas on the surface. Figure 4a displays the morphology and chemical composition of Co@CPA through a microscopic map of the selected area. The hierarchical image in (b) reveals a relatively uniform distribution of elements such as Co, C, N, and P. Furthermore, elements Co, C, N, and P are individually represented in the distribution maps (c), (d), (e), and (f) for this specific area. The mapping element analysis results reveal a uniform distribution of Co, C, N, and P elements across the system, suggesting effective coordination between Co and CPA. This led to the formation of a uniform Co@CPA structure with clear hierarchy.

**Figure 5:**
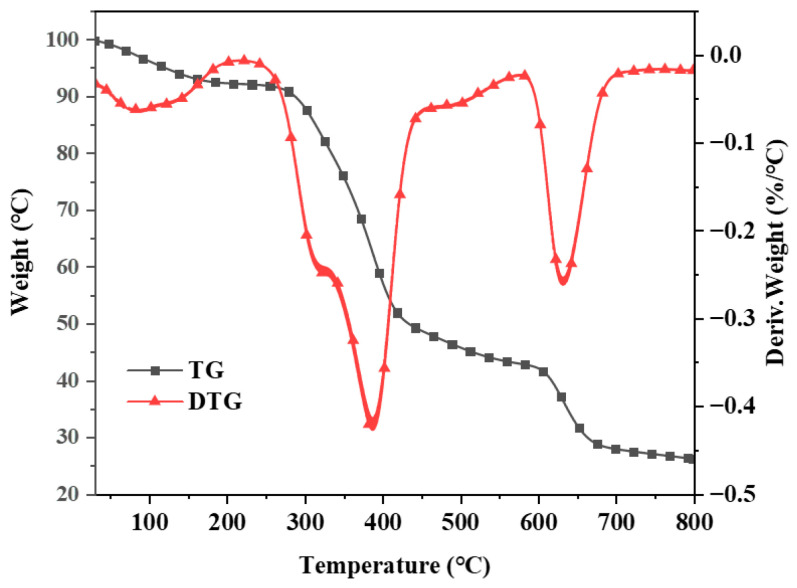
TGA and DTG curves of Co@CPA.

The TG and DTG curves were generated in a high-purity nitrogen atmosphere with a heating rate of 10 °C/min (Figure 5). The TG curve indicates that the thermal degradation of Co@CPA can be categorized into three main stages. The initial stage at 100 °C is attributed to the vacuum drying of Co@CPA, with weight loss likely due to the evaporation of absorbed water molecules. The second stage, occurring between 300 and 400 °C, corresponds to the primary thermal decomposition process of the Co@CPA compound, possibly resulting from the breakage of chemical bonds between CPA and Co metal. The third stage, observed between 400 and 650 °C, signifies the completion of the thermal degradation process of Co@CPA. Notably, the residual carbon content at 800 °C is 35.48%. These findings provide additional evidence that CPA and Co elements are effectively coordinated, forming a complex with a distinct heat difference value.

### 3.3. Performance Testing of TPU and Its Composite Materials

Table 2 presents the ultimate oxygen index and vertical combustion data for TPU and its composite materials. The data reveal that pure TPU material has an LOI value of 18% and did not achieve any classification in the UL-94 test. During testing, a substantial number of molten droplets were produced, leading to ignition of the degreased cotton below, accompanied by a significant emission of black smoke. Upon testing with a certain quantity of Co@CPA, the LOI value of the TPU composite material increased to 19.5%, still not attaining a UL-94 grade, but with a notable reduction in droplet formation. Furthermore, the LOI value of the TPU composite material containing ODOPB additive was only 20%.

The addition of a single flame retardant did not achieve the desired effect on TPU. However, the composite flame retardant consisting of 12% Co@CPA, 12% ODOPB, and 1% SiO_2_ significantly increased the LOI value of TPU and achieved a V-0 level in the UL-94 test. When the ratio of Co@CPA to ODOPB was 1:2, the LOI of the composite material reached 26.5% and achieved V-0 level without dripping. Compared to pure TPU materials, the limiting oxygen index increased by 38.9% and 47%. This improvement is attributed to the metaphosphoric acid produced by the thermal decomposition of Co@CPA and ODOPB during combustion, which enhances dehydration and carbonization of the char-forming agent. The metal oxides generated cover the material’s surface, preventing the transfer of heat and combustible substances, thus enhancing the material’s combustion performance. The cobalt ions exposed after the Co@CPA skeleton collapse catalyze the TPU matrix into carbon, and the porous metal oxide (Co_3_O_4_) formed during combustion inhibits the exchange of oxygen and degradation products, enhancing the flame-retardant performance of the TPU composite material.

**Figure 6:**
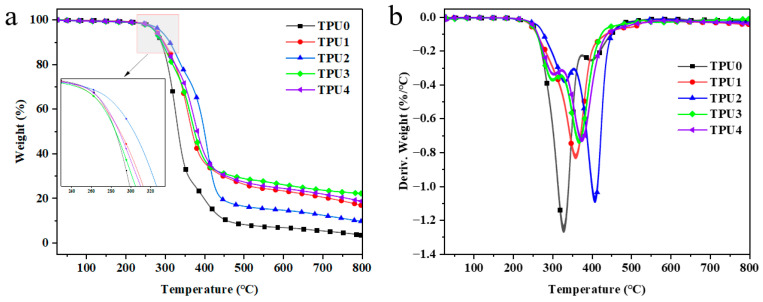
TGA (**a**) and DTG (**b**) curves of TPU and TPU composites.

In order to further study the impact of Co@CPA and ODOPB on the thermal stability of TPU, thermogravimetric analysis was conducted to assess the stability of TPU and its composites at elevated temperatures. Figure 6 illustrates that pure TPU undergoes one-step degradation at high temperatures, with an initial decomposition temperature (T*_−5wt%_*) of 275.25 °C and complete decomposition at 800 °C, leaving almost no residual carbon. Both the TG curves of TPU composites and pure TPU display one-step degradation, showing similar decomposition behavior. Table 3 reveals that the addition of Co@CPA results in a slight increase in the initial decomposition temperature (T*_−5wt%_*) and maximum weight loss temperature (T*_max_*) of TPU composites, with T*_max_* ranking as TPU2 > TPU4 > TPU3 > TPU1 > TPU. At 800 °C, TPU3 shows a 524.1% increase in residual carbon compared to pure TPU, while TPU4 increases by 451.2%, indicating the high thermal stability and catalytic carbonization effect of Co@CPA in combination with ODOPB. These findings suggest that a 1:2 ratio of Co@CPA to ODOPB significantly enhances the thermal stability of TPU, surpassing pure TPU in terms of thermal degradation rate and carbonization.

**Figure 7:**
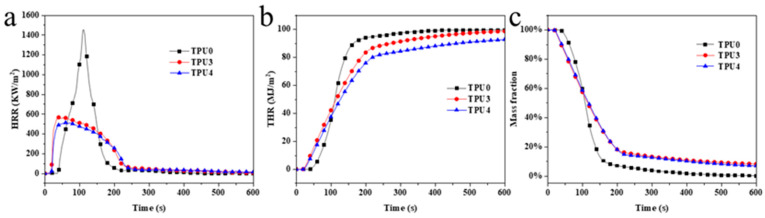
(**a**) HRR, (**b**) THR and (**c**) weight loss ratio curves of TPU and TPU composites.

The HRR (heat release rate), THR (Toal Heat Release), and thermal weight loss curves of TPU composite materials are illustrated in Figure 7, with specific data provided in Table 4. Pure TPU exhibited violent burning at 38 s, reaching a pk-HRR value of 1453.6 kW/m^2^, THR of 99.59 kW/m^2^, MARHE (maximum average heat release rate) of 570.32 kW/m^2^, av-COY of 0.33 kg/kg, and av-CO_2_Y of 7.55 kg/kg. The inclusion of 12% ODOPB and 12% in TPU3 Co@CPA with 1% SiO_2_ led to a pk-HRR value of 304.95 kW/m^2^, THR of 98.91 kW/m^2^, and MARHE of 438.62 kW/m^2^. These values represented a 79%, 0.68%, and 23% increase, respectively, compared to pure TPU. Flame retardants were observed to play a significant role in the combustion process of materials. With 16% ODOPB and 8% in TPU4 Co@CPA, the values of 1% SiO_2_, the pk-HRR, THR, MARHE, av-COY, and av-CO_2_Y decreased significantly to 318.38 kW/m^2^, 93.05 kW/m^2^, 392.82 kW/m^2^, 0.20 kg/kg, and 3.86 kg/kg, respectively. Compared to pure TPU, these values decreased by 78.1%, 6.6%, 33.1%, 39.4%, and 48.9%, respectively.

Test results indicated that TPU4 exhibited lower total heat release and average effective combustion heat compared to TPU3, with reduced average yields of CO and CO_2_. TPU4 demonstrated a positive impact on the heat release of toxic smoke in comparison to TPU3. Further evaluation of the fire safety of TPU included the calculation of MARHE, revealing a significant reduction in MARHE value for TPU4 compared to pure TPU and TPU3. This suggested that increasing the proportion of ODOPB in Co@CPA was associated with slowing down the combustion rate and enhancing the flame-retardant properties of TPU composite materials. Based on the flame-retardant performance tests conducted, it can be concluded that the TPU4 formula sample exhibited the most effective flame-retardant effect on TPU composite materials.

**Figure 8:**
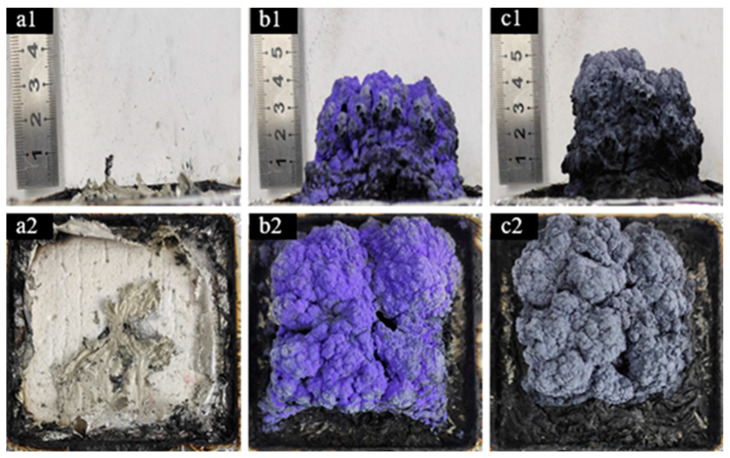
Photo of carbon residue after CCT: (**a1**,**a2**) TPU0, (**b1**,**b2**) TPU3, and (**c1**,**c2**) TPU4.

The macroscopic morphology of residual carbon after cone calorimetry testing is illustrated in Figure 8, showing the residual carbon of TPU and its composite materials. In the image, pure TPU is shown to have burned completely with minimal residual carbon, while the addition of Co@CPA to TPU composite materials containing ODOPB and SiO_2_ resulted in a significant increase in residual carbon thickness post cone calorimetry testing. Figure 8b1,b2 demonstrate that the residual carbon of TPU3 composite material, with a 1:1 ratio of ODOPB addition, is coated with a blue substance on the surface, identified as CoSiO_3_, indicating a synergistic effect between ODOPB, SiO_2_ and Co@CPA during TPU combustion. On the other hand, Figure 8c1,c2 reveal that TPU4 composite material, with a 1:2 ODOPB addition ratio, exhibits a denser and harder residual carbon layer post-combustion, acting as an effective physical barrier to heat, oxygen, and combustible gases within the TPU matrix for improved flame retardancy.

**Figure 9:**
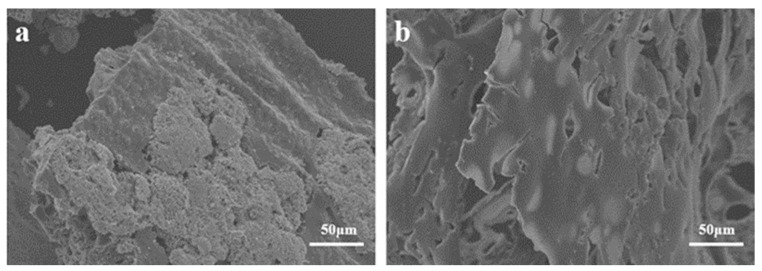
SEM images of the char residues after CCT for TPU3 (**a**) and TPU4 (**b**).

The SEM image in Figure 9 displays residual carbon in TPU composite material following a cone calorimetry test, revealing the micro surface of the carbon layer. In Figure 7a, the carbon layer surface of TPU3 composite material exhibits some agglomerates and lacks a dense structure. In contrast, the carbon layer surface of TPU4 composite material in Figure 9b appears denser and smoother, both providing insulation and effectively preventing the exposure of combustible gases. Therefore, the relationship between ODOPB and Co@CPA indicates that an increased proportion can enhance the flame retardancy of TPU.

**Figure 10:**
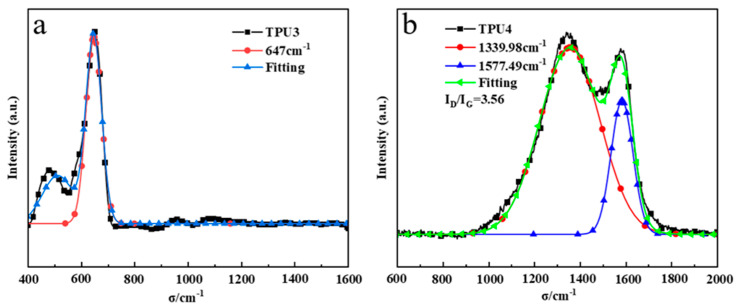
Raman spectra of the char residues from TPU3 (**a**) and TPU4 (**b**).

The D peak (1350 cm^−1^) is an absorption peak resulting from defects in the carbon atom lattice, while the G peak (1580 cm^−1^) is formed by in-plane stretching vibration of carbon atom sp2 hybridization. The I_D_/I_G_ ratio represents the peak intensity relationship between the D peak and the G peak. Thermogravimetric and cone calorimetry analysis of TPU suggests that pure TPU leaves minimal residual carbon post-high-temperature thermal decomposition. Figure 10a,b illustrate the addition of 12% ODOPB and 12% Co@CPA to TPU residue with 1% SiO_2_, as well as the addition of 16% ODOPB and 8% Co@CPA to TPU residue with 1% SiO_2_. The Raman spectrum of TPU residual carbon in Figure 10a displays a strong peak at 400–700 cm^−1^, characteristic of Co_3_O_4_ crystal Raman. In Figure 10b, TPU4 exhibits an I_D_/I_G_ value of 3.56, indicating higher carbon layer graphitization with lower values. This suggests that a 1:1 ratio of Co@CPA to ODOPB is not conducive to TPU carbonization, while the addition of ODOPB promotes TPU carbonization. ODOPB, in conjunction with Co@CPA, can synergistically enhance the flame retardancy of TPU materials.

**Figure 11:**
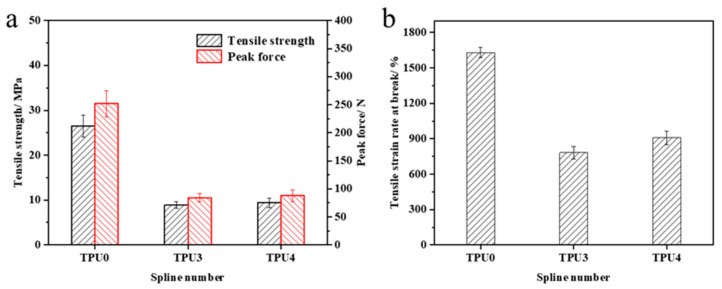
Mechanical properties of TPU and TPU composites (**a**) Tenslle strength, (**b**) Tenslle strain rate at break.

The mechanical property test results of TPU and its composite materials are illustrated in Figure 11. Pure TPU exhibits a tensile strength of 26.5 MPa, a peak force of 252.1 N, and a tensile strain rate at break of 1631.1%. The incorporation of flame retardants affects the material’s mechanical properties to some extent, resulting in reductions in tensile strength, peak force, and tensile strain rate at break for the composite material. The magnitude of this reduction diminishes with higher ODOPB content. This phenomenon is primarily due to the embrittlement caused by the introduction of metal elements, which ODOPB mitigates. Research findings indicate that optimal mechanical properties of the TPU composite material are achieved when the total flame-retardant content is 25% and the ODOPB to Co@CPA ratio is 2:1. Furthermore, the material exhibits good flame-retardant properties, expanding its potential applications.

## 4. Conclusions

A cobalt-containing organometallic complex, Co@CPA, was successfully synthesized and found to enhance the flame-retardant properties of TPU. The optimal flame-retardant effect was achieved at a TPU:ODOPB:Co@CPA:SiO_2_ ratio of 75:16:8:1, resulting in a LOI value of 26.5% and UL-94 V-0 rating. Compared to pure TPU, the composite material showed reductions of 6.6% in total heat release, 39.4% in CO production, and 48.9% in CO_2_ production. Residual carbon analysis indicated a denser microstructure in the TPU composite with flame retardants. The char residue layer exhibited a high degree of graphitization, effectively protecting the matrix. Overall, a synergistic effect was observed in Co@CPA in reducing heat release, inhibiting smoke generation, and promoting the formation of the char residue layer. This effect has been proven to enhance the flame retardancy of TPU.

The study focuses on a single metal-based cyclic phosphoronitrile compound that must be combined with other compounds to effectively act as a flame retardant. Future research should investigate the varying flame-retardant effects of different metal-based cyclic phosphoronitrile compounds (e.g., Mn, Sb, Zn) on diverse materials. Our research findings suggest that Co@CPA exhibits outstanding performance, with potential for further expansion in application areas. Different metal-based cyclic phosphoronitrile compounds hold significance in enriching phosphorus-based fine chemicals.

## Figures and Tables

**Table 1:** Formula of TPU and TPU composite materials.

Num.	w(TPU)/%	w(Co@CPA)/%	w(ODOPB)/%	w(SiO_2_)/%
TPU0	100	N/A	N/A	N/A
TPU1	88	12	N/A	N/A
TPU2	75	N/A	25	N/A
TPU3	75	12	12	1
TPU4	75	8	16	1

**Table 2:** Flame retardancy test of TPU and TPU composites.

Num.	Flame-Retardant Performance Testing		
	LOI/%	UL-94	Molten Drop
TPU0	18.0 ± 0.2	NR	Yes
TPU1	19.5 ± 0.2	NR	Yes
TPU2	20.0 ± 0.1	NR	Yes
TPU3	25.0 ± 0.3	V-0	No
TPU4	26.5 ± 0.1	V-0	Yes

Note: NR represents no level and cannot pass UL-94 testing.

**Table 3:** Thermogravimetric test data of TPU and TPU composite materials.

Sample	T*_−5wt%_*/°C	T*_max_*/°C	Residue at 800 °C/wt%
TPU0	275.25	328.03	3.57
TPU1	275.25	358.18	16.86
TPU2	288.93	407.25	9.65
TPU3	273.67	367.27	22.15
TPU4	276.89	374.00	19.68

**Table 4:** The cone calorimeter data of TPU and TPU composites.

Num.	TTI/	THR/	pk-HRR/	av-COY/	av-CO_2_Y/	av-EHC/	MARHE/
	s	(MJ/m^2^)	(kW/m^2^)	(kg/kg)	(kg/kg)	(MJ/kg)	(kW/m^2^)
TPU0	38	99.59	1453.6	0.33	7.55	27.43	570.32
TPU3	20	98.91	304.95	0.23	4.96	29.44	438.62
TPU4	20	93.05	318.38	0.20	3.86	27.31	392.82

## Data Availability

The data presented in this study are available on request from the corresponding author.
